# Adolescent Patient's Perceptions During Treatment With Class II Elastics

**DOI:** 10.1155/2024/1489397

**Published:** 2024-08-06

**Authors:** V. Bogdanov, D. Ilova, Gr. Yordanova

**Affiliations:** Department of Orthodontics, Faculty of Dental Medicine, Medical University, Sofia, Bulgaria

**Keywords:** Class II malocclusion, elastics, mandibular advancement, orthodontics, TMD

## Abstract

The article presents a case of a 13-year-old adolescent male patient who started orthodontic treatment at the age of 12. Before treatment, he was diagnosed with narrow maxilla, proclination of upper incisors, deep overbite, distal occlusion bilaterally with significant sagittal overjet in frontal area, skeletal Class II, and hypodivergent growth pattern. During treatment, the patient is in his pubertal growth spurt. About 2 months after intermaxillary Class II elastics (1/4 heavy, 6.5 Oz) were applied, he complained of pain during mastication, wide opening of the mouth, and sometimes during protrusive and lateral movements in the right TMJ. The TMJ X-ray examination did not reveal abnormal morphological changes. Occlusion was evaluated by an electromyographic device, Teethan. The result was typical for Class II malocclusion. During the bilateral palpation of the zones of TMJ and opening of the mouth and chewing, the patient reported pain on the right side. There was no clicking in the joint. The elastic wear was stopped, and soon afterwards, the pain disappeared. These complaints point to a possible relationship between orthodontic treatment and TMJ pain. However, the disappearance of complaints after the removal of the Class II elastics points that the temporomandibular joint disorder (TMD) symptoms are reversible and resolved.

## 1. Background

Temporomandibular joint disorder (TMD) is considered multifactorial, caused by different factors (trauma, anatomical, pathophysiological, and psychosocial factors), which, according to studies, are present in nearly 70% of the population of different age groups [1]. The main clinical signs and symptoms of TMD are clicking of the joint, abnormal mandibular movement, limited mouth opening, and pain in the joint and the face muscles [2]. This disorder is often found in children and adolescents, and pain during palpation of the chewing muscles and click of the TMJ are common [3]. It is generally accepted that one of the main etiological factors for TMD is occlusion. According to different authors, the most common disorders associated with TMD are deviations of the occlusal interrelations, Angle Classes II and III, severe overjet, anterior open bite, and posterior crossbite [1, 3]. Because correction of abnormal occlusion is an object of orthodontic treatment, some studies report that symptoms and signs of TMD occasionally occur during or after orthodontic treatment [4], as TMJ and condylar cartilage are very sensitive to mechanical stress that may cause its degradation and even dislocation. Orthodontic treatment is a long-lasting process applied mainly in young age; the above-mentioned complications of the TMJ involve mostly children and adolescents. Because of the different malocclusions and different orthodontic appliances used for treatment, the data concerning the relationship between orthodontic treatment and incidence of TMD are still contradictory. Some researchers consider that there is no significant evidence for the association between TMD and orthodontic treatment [5].

Intermaxillary elastics are widely used to apply orthodontic forces during orthodontic treatment. Usually, the force is applied for approximately 24 h а day and is expected to achieve remodeling of the dentoalveolar bone. In animal models, intermaxillary elastics show mechanical effects on the TMJ by exerting stress on the TMJ disk in a normal position and in an anterior position with Class II and Class III elastics [6]. A recent comprehensive study of a three-dimensional biomechanical TMJ model analyzed whether variations of intermaxillary elastics influence the movement of the mandible and conduct excessive mechanical stress, thus developing harmful effects in the healthy TMJ, and if so, will this effect be consistent with the aim of the therapeutic plan? The study showed displacement of the mandible with intermaxillary elastics during mouth opening and closure. With asymmetrical elastics at the early stage of opening, the mouth is pulled to the anchorage side but this effect is compensated by more muscle force. The results show that the stress on the disk and cartilage with elastics is not higher than without elastics. Therefore, the authors consider that medium force elastics do not have a deleterious effect on a healthy TMJ [2].

Lan Ding et al. [7] summarized the results of functional mandibular advancement (FMA) on the temporomandibular joint and possible side effects and incidence of temporomandibular disorders in adolescent patients. Only 80 out of 579 observed individuals developed temporomandibular symptoms during and after treatment, which disappeared during follow-up time. The statistical outcomes proved that patients who received FMA did not show higher tendency to develop temporomandibular symptoms, so FMA will not have side effects on TMJ of adolescent patients.

In a computer-aided model, Gurbanov, Baz, and Öz [6] simulated the effects of Class II and Class III elastics. They found that Class II elastics create greater stress on the TMJ structures than Class III elastics. The authors concluded that the elastic force applied during fixed orthodontic treatment increases stress, especially in Class II patients. If the disc is in an anterior position, the stresses could be more harmful to the retrodiscal tissue. Moreover, the authors recommended that during orthodontic treatment, the TMJ should be carefully assessed to avoid irreversible damage.

## 2. Case Description

We present a 12-year-old patient seeking orthodontic treatment for impaired occlusion and aesthetics. The parents reported interceptive treatment at the age of nine with an expansion plate with a tongue barrier and an oral habit—thumb sucking until that age.

**Figure 1:**
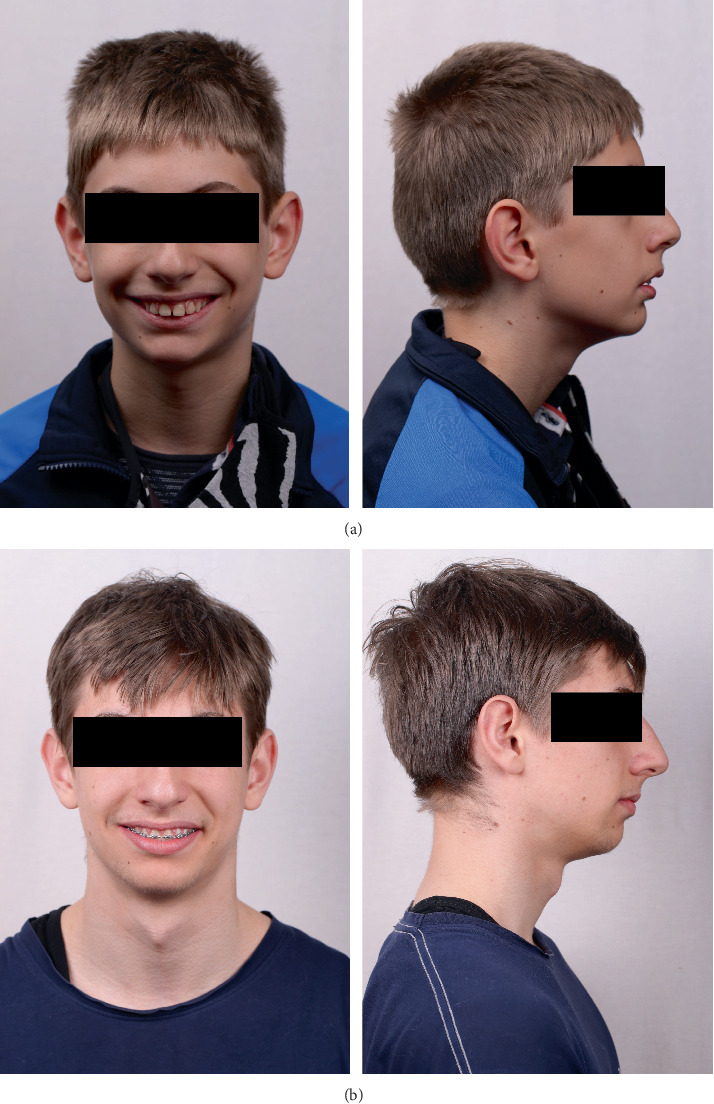
(a) En face and profile view before treatment. (b) En face and profile view during treatment—2 years later.

**Figure 2:**
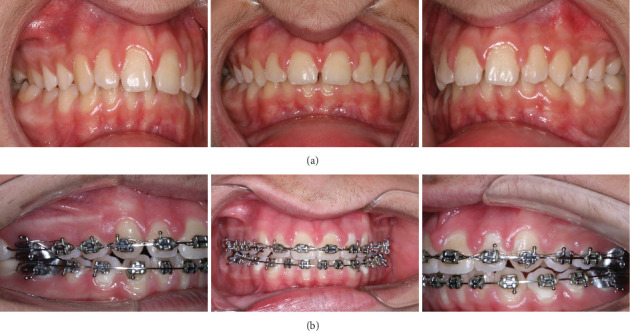
(a) Intraoral photos before treatment. (b) Intraoral photos 2 years after start of treatment.

Extraoral examination before treatment revealed convex profile, retrognathic chin, shortened lower third of the face, proclined upper incisors, and incompetent lips (Figure 1(a)). Intraoral examination showed constricted maxilla, proclination of the upper anterior teeth, crossbite in first molars on the right, deep overbite, and distal occlusion with overjet of approximately 8 mm (Figure 2(a)). Usual clinical tests were performed to examine the basic functions and tone of individual muscle groups. TMJ was examined by palpation both in front of the ear and into the auditory meatus. Masticatory muscles were palpated, and no pain or tenderness was reported. During opening and closing, there was no clicking or deviated path of movement of the mandible.

On plaster models, the diagnosis was confirmed: narrow maxilla, proclination of the upper incisors, and Class II occlusion.

**Figure 3:**
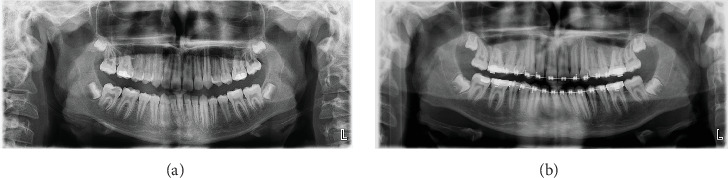
Panoramic X-rays (a) before and (b) 2 years after the onset of treatment.

Panoramic X-ray before and 2 years after onset of treatment showed that all the permanent teeth are present, the bone density and structure are normal, and there are no signs of any morphological alterations in the TMJ structures (Figures 3(a) and 3(b)).

**Figure 4:**
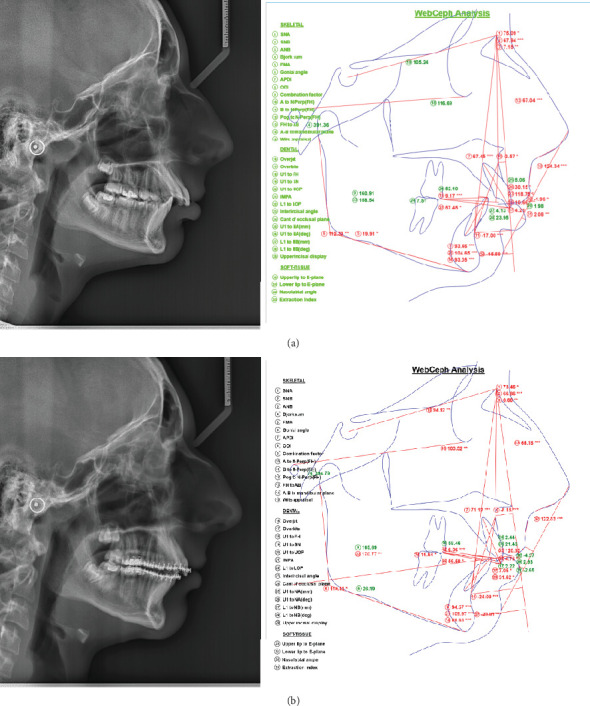
(a) Cephalometric analysis and data before treatment. (b) Cephalometric analysis and data after 2 years of treatment.

Lateral cephalometry confirmed skeletal Class II, and proclination of upper and lower incisors, hypodivergent pattern (Figure 4), and skeletal maturation were evaluated as CVM 2-3.

**Figure 5:**
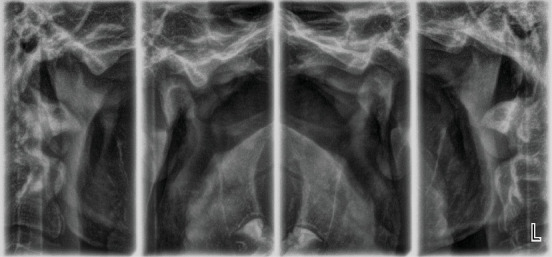
TMJ X-ray.

Based on the radiological and biometric findings, a treatment plan was developed in two main stages: leveling and aligning the arches and a second stage correction of the occlusal relationships by mesialisation of the mandible by intermaxillary elastics. The parents signed an informed consent to conduct orthodontic treatment and use the documentation from it for scientific purposes without revealing their identity. Orthodontic treatment with a preadjusted appliance (prescription MBT, slot 0.022 x 0.028) was performed. Leveling and alignment took 5 months, and 0.017 x 0.025 stainless steel archwires were installed. Attached to lower first molars and upper canines, intermaxillary Class II elastics (1/4⁣^″^, 4.5 Oz) were prescribed for a period of 4 weeks. The patient was instructed to wear them full time except for eating and brushing and to change them every 12 h. As the patient is skeletal Class II, heavier forces were applied. Eight weeks after the use of heavy (1/4″, 6.5 Oz) intermaxillary elastics, the patient complained of pain during mastication, wide opening of the mouth, and sometimes during protrusive and lateral movements in the right TMJ. Palpation of the masseter muscle and TMJ area on the right side showed pain and tenderness. Mouth opening was unimpeded; the pathway of the opening and closing was unaltered. The X-ray of the TMJ did not show any morphological alterations (Figure 5).

As the complaints disappeared, a second TMJ X-ray was not assigned.

**Figure 6:**
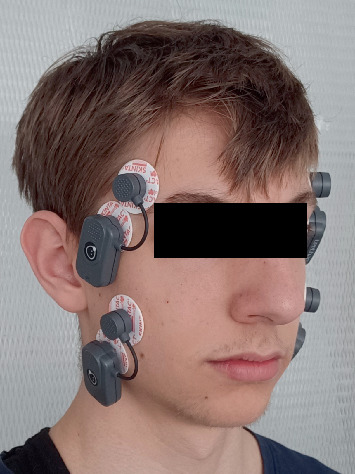
The patient with the EMG probes adhered to the skin.

A surface electromyography (sEMG) evaluation was done by Teethan (Teethan S.p.A., Italy) appliance. Тhe purpose of the sEMG study was to determine if there was any muscle imbalance due to occlusal interference. It was done immediately after the beginning of the complaints of pain in the TMJ and 1 year later. The recordings were taken bilaterally from masseter and anterior temporalis muscles during a 5-s maximal voluntary clench (Figure 6).

**Figure 7:**
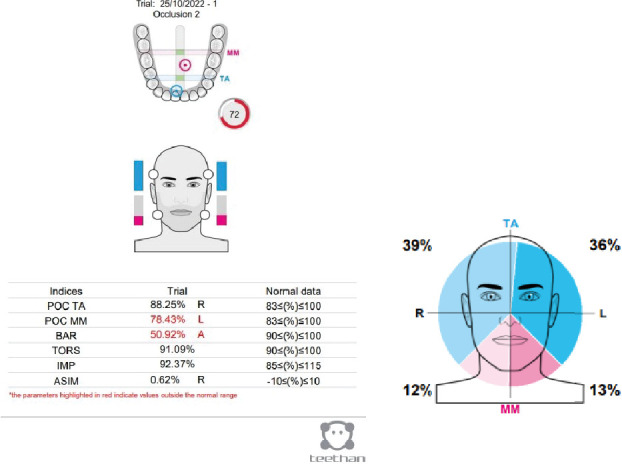
Electromyographic evaluation of the occlusion.

The results show that on the left side, there were tighter contacts between the molars, and the barycenter (BAR) is displaced anteriorly than the norm. The latter is typical for Class II malocclusions, and prevalence of the temporal muscles. There was no functional shift or torsion (TORS) which is the predisposition for TMD, or decrease of the muscle force (IMP), which results from a TMD-related pain (Figure 7).

After the appearance of the complaints, the Class II therapy with elastics was immediately stopped. Oral nonsteroidal anti-inflammatory drug therapy was prescribed. Soft diet and foods easy to chew were recommended as well. After a period of 1 month, complaints subsided thoroughly. The elastic wear then restarted with 1/4⁣^″^ medium, 4.5 Oz.

Two years after the onset of orthodontic treatment, improvement of the patient's condition was observed. The extraoral examination revealed improvement of the facial proportions, especially the profile (Figure 1(b)). Intraoral examination showed improvement in the occlusion (Figure 2(b)). The panoramic X-ray observed has no signs of morphological alterations in the TMJ 2 years after the beginning of the treatment (Figures 3(a) and 3(b)). Cephalometric analysis 2 years after the onset of the treatment showed significant improvement of the inclination of the upper incisors (Figures 4(a) and 4(b)). This was achieved by 18 months of constant wearing of elastics. The patient is still in treatment, and the elastic wear continues.

## 3. Discussion

Usually, no significant orofacial pain or undesirable change in the mouth opening range occurs because of orthodontic elastic treatment. If any, they are usually transitory and with moderate intensity. TMD is not induced due to orthodontic treatment [8]. The link between occlusal changes and TMD has been a subject of interest and investigation for a long time [9]. A weak association between occlusal problems and TMD is suggested [10, 11]. A study conducted by Giray and Sadry [12] aimed to observe changes in tooth movements of patients with Class I and Class II malocclusion during the first 6 months of orthodontic treatment in order to investigate the relation between TMJ problems and these changes. The authors conclude that contrary to the prevalent opinion in the literature, no significant relationship was observed between the first 6-month period of orthodontic treatment and TMD. In our case due to the immediate response to the pain, the patient recovered quickly and without any consequences.

The examination of children 12–18 years of age with malocclusion and treated with fixed orthodontic appliances was compared with children with malocclusion. The most common symptom of TMJ clicking sound was found in 56.4% in orthodontic patients and in 46.6% in the control group respondents. A significantly higher percent of female respondents in both groups experienced headache problems (*p* < 0.03). The authors concluded that there is no correlation between fixed orthodontic treatment and development of signs and symptoms of TMD [13]. The dysfunction of TMJs and their adjacent structures are manifested with pain in the area of TMJs and masticatory muscles, and TMJ sounds as a symptom of structural changes and limited mobility of the lower jaw [14].

Occlusal disorders were considered as one of the major etiological factors for TMD. Recently however, the role of occlusion in TMD is a controversial issue. The concepts of this correlation are recommended occlusal splints, anterior repositioning splint, occlusal adjustment, etc. In contrast, various dental interventions, including orthodontic treatment, are considered possible causes of TMD without real evidence [15]. New scientific research suggests that TMD is a multifactorial disorder with complex etiology and pathophysiology [16].

A case report by Alajbeg I. et al. [17] presented a young woman complaining of myofascial pain and sudden occlusal change (anterior open bite), occurring shortly after the administration of a soft night guard. MRI of TMJs excluded disc displacement, and the final diagnosis was myalgia of the masseter and temporalis muscles. The patient received a combined treatment including pharmacotherapy and a stabilization splint. After TMD symptoms resolved, the patient was diagnosed with skeletal Class II retrognathic face and was subjected to orthodontic treatment by a fixed appliance with vertical intermaxillary elastics. In similar cases, the authors recommend orthodontic therapy, which should be applied only when the TMD pain resolves. We find similar complaints to the patient described by us. In our case, after the patient complained of pain on the right side, the Class II elastic was immediately stopped. One month later, complaints subsided thoroughly and the elastic wear was resumed with 1/4⁣^″^ medium, 4.5 Oz.

Analysis of reported studies showed that after treatment with functional appliances, the condyle was found to be in a more forward position with remodeling of the condyle and adaptation of the morphology of the glenoid fossa [18]. No significant adverse effects on the TMJ were observed in healthy patients, and the appliances could improve joints that initially presented forward dislocation of the disc. In cases with mandibular retrognatism during the first stage of orthodontic treatment, it is necessary to remove the blockages in the path of medialization of the lower jaw. The aim is by bringing the condyles forward and downward to stimulate mandibular growth [19]. Many authors affirm that treatment with these devices does not affect the incidence of TMD [20, 21]. Another point of interest is the consequences of these devices on the TMJ in subjects with pre-existing disorder [21]. Some authors found that functional appliances cause stretching of the retrodiscal tissue [22, 23]. Others [21] do not find significant changes in the shape or the displacement of the disk after 12 months of use of Herbst appliance in adolescents. In patients with prior disk displacement, the disk was not recaptured [20]. Other authors however found an improvement of disk position compared to the initial disk displacement [24, 25]; others reported no adverse effects on the articular disk morphology [20]. In single patients, the disk changed from biconcave to nonbiconcave [21]. A case-control study with Herbst appliance found adaptive displacement of the condyles and consequent remodeling of the glenoid fossa compared to the control group, treated with fixed appliances and Class II elastics [19]. By MRI, Wadhawan et al. [22] found that the condyle and disk were displaced during treatment but returned to their initial position when the treatment ended. Changes in condyle position are not clinically significant [26], and anteroinferior displacement of the glenoid fossa [25], or no remodeling of the fossa [26], is found as a reaction to Herbst treatment. Among other changes, restricted maxillary growth (Herbst appliance) [19], increased volume of the condyle, and the length of the mandible–Twin Block treatment [27] can be mentioned; however, no adverse effect can be noted on the TMJ in healthy subjects. Conti et al. [28], in a cross-sectional study, showed a mild and moderate association between orthodontic treatment and TMD.

## 4. Conclusion

Orthodontic treatment is rarely а cause for TMD, especially in growing patients. Nevertheless, the forces applied for correction of the occlusion should be light. When there are complaints, thorough diagnosis, including examination of the joints and the occlusion, is necessary. Soft diet and pause in using elastics can be helpful for the patient in case the TMD occurs during orthodontic treatment. Nonsteroidal anti-inflammatory drugs can help for relieving the pain in the acute period. Careful monitoring of the patient and stepwise application of forces can be beneficial.
